# Developing a method for customized induction of flowering

**DOI:** 10.1186/1472-6750-11-36

**Published:** 2011-04-11

**Authors:** Chin Chin Yeoh, Martin Balcerowicz, Rebecca Laurie, Richard Macknight, Joanna Putterill

**Affiliations:** 1 Plant Molecular Sciences, School of Biological Sciences, University of Auckland, Private Bag 92019, Auckland, New Zealand; 2 Department of Biochemistry, University of Otago, PO Box 56, Dunedin 9054, New Zealand

## Abstract

**Background:**

The ability to induce flowering on demand is of significant biotechnological interest. FT protein has been recently identified as an important component of the mobile flowering hormone, florigen, whose function is conserved across the plant kingdom. We therefore focused on manipulation of both endogenous and heterologous *FT *genes to develop a floral induction system where flowering would be inhibited until it was induced on demand. The concept was tested in the model plant *Arabidopsis thaliana *(Arabidopsis).

**Results:**

Our starting point was plants with strongly delayed flowering due to silencing of *FT *with an artificial microRNA directed at *FT *(*amiR-FT*) [1]. First, we showed that constitutive expression of a heterologous *FT *gene (*FTa1*), from the model legume *Medicago truncatula*, (Medicago) was able to rescue the *amiR-FT *late-flowering phenotype. In order to induce flowering in a controlled way, the *FTa1 *gene was then expressed under the control of an alcohol-inducible promoter in the late flowering *amiR-FT *plants. Upon exposure to ethanol, *FTa1 *was rapidly up regulated and this resulted in the synchronous induction of flowering.

**Conclusions:**

We have thus demonstrated a controlled-inducible flowering system using a novel combination of endogenous and heterologous *FT *genes. The universal florigenic nature of FT suggests that this type of system should be applicable to crops of economic value where flowering control is desirable.

## Background

Flowering time is an important plant breeding target [reviewed by [2]]. The time at which flowering occurs affects the ensuing success of flower, seed and fruit development, ease of harvest and marketing. In addition, since flowering of vegetative crops and forages can be negatively correlated with yield and nutritive quality, the ability to delay flowering in such plants would be advantageous. Therefore, our goal is to develop molecular-genetic tools for customization of flowering in economically-important plants.

Plants use a combination of environmental and developmental cues to trigger flowering. The genetic networks that are involved in perception and response to these floral signals is best worked out in the model plant *Arabidopsis thaliana *(Arabidopsis) [3-8]. Many of the flowering time pathways ultimately converge on a set of genes called floral integrators, which includes *FLOWERING LOCUS T *(*FT*) and a closely related and partially redundant homologue *TWIN SISTER OF FT *(*TSF*) [9-12]. FT strongly activates flowering in Arabidopsis and mutants lacking a functional *FT *gene flower very late, while transgenic plants over expressing *FT *flower much earlier than wild type plants [9,10].

*FT *is the primary target of several flowering time pathways. These include the long day pathway which promotes flowering in response to long day photoperiods via CONSTANS (CO) mediated up regulation of *FT*, and the vernalisation and autonomous pathways that function to down regulate the flowering repressor *FLOWERING LOCUS C *(*FLC*) thereby alleviating FLC's repression of *FT *[reviewed by [6,7]]. *FT *transcript is expressed in the leaf vasculature where FT protein is produced and moves via the phloem to the shoot tip [1,13-15]. In the shoot apical meristem, FT partners with FD, a bZIP transcription factor and switches on genes, such as *APETALA1*, to initiate floral development [16,17]. Thus, FT protein functions as an important component of the mobile flowering hormone, florigen [see reviews by [6,8]].

**Figure 1:**
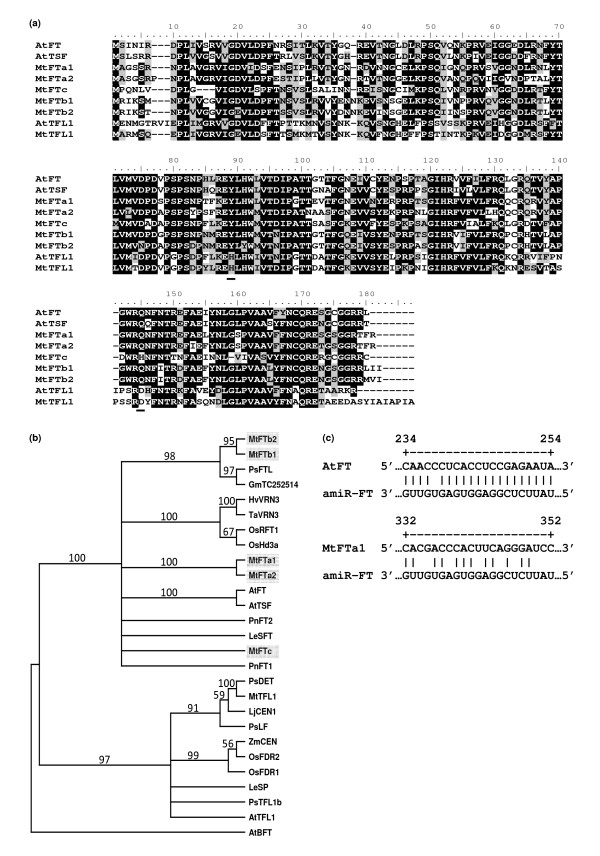
**Protein and mRNA sequence alignments and phylogenetic analysis of FT and TFL1 proteins**. a) Alignment of selected full-length FT and TFL1 proteins from Medicago and Arabidopsis. Background black shading indicates identical amino acids shared in 50% or more sequences, gray indicates similar amino acids shared in 50% or more sequences. Black underlining marks key functional amino acid residues in FT (see text for details). b) Neighbour joining tree of selected FT and TFL1 full length proteins from 11 different plant species. The tree was rooted on AtBFT. Bootstrap values from 1000 replications are shown as a percentage above each branch. The Medicago proteins are highlighted with a gray box. c) Alignment of the sequence of the *amiR-FT *to Arabidopsis *FT *transcript (above) and to Medicago *FTa1 *mRNA (below). At *Arabidopsis thaliana*; Gm *Glycine max*, Hv *Hordeum vulgare*; Le *Lycopersicon esculentum *now *Solanum lycopersicum*; Lj *Lotus japonicus*, Mt *Medicago truncatula *Os *Oryza sativa*, rice, Pn *Pharbitis nil*, Ps *Pisum sativum*, Ta *Triticum aestivum*, Zm *Zea mays*. Gene identifiers (At) or Accession numbers are listed in the methods.

Older physiological studies demonstrated the universal nature of florigen in plants [reviewed by [18]]. Consistent with this, orthologues of the Arabidopsis *FT *gene are widespread in the plant kingdom (Figure 1a, b) and promote flowering even in plants with different day length requirements to Arabidopsis. For example, the tomato *FT *orthologue *SINGLE FLOWER TRUSS *(*SFT*) promotes flowering in day neutral tomato [19] and two rice *FT *orthologues, *Heading date 3a *(*Hd3a*) and *RICE FLOWERING LOCUS T 1 *(*RFT1*) promote flowering in rice, a short-day plant [20,21]. Like Arabidopsis *FT*, the *SFT*, *Hd3a *and *RFT1 *genes encode a graft transmissible floral signal [19,22,23]. In addition, over expression of *FT *orthologues such as *SFT *can promote flowering in heterologous transgenic plants [19]. Thus, despite differences in the upstream signaling pathways in different plants, the induction of FT expression in leaves and its movement to the apex where it triggers flowering appears to be conserved.

Genetic engineering of flowering time in many cultivated species has been achieved by manipulation of floral repressors or floral promoters, including the over expression or inducible expression of *FT *to accelerate flowering in transgenic plants [2,24,25]. Because of the conservation of FT function across the plant kingdom, we have focused on developing a controlled-inducible flowering system that manipulates endogenous and heterologous *FT *genes. This utilizes the induction of a heterologous *FT *gene to trigger flowering in a background in which flowering has been inhibited using an artifical microRNA that targets the endogenous *FT *gene. The system is based on the idea that artificial microRNAs can be designed to specifically down regulate an endogenous gene, but should not affect the expression of a sufficiently divergent functional orthologue from another species. Here, we report on the development of this system for inducing flowering on demand and demonstrate its utility in Arabidopsis.

## Results and Discussion

### Selecting a heterologous *FT *gene to overcome the late flowering phenotype of *amiR-FT *Arabidopsis plants

Our starting point was a late flowering transgenic line of Arabidopsis in which flowering was inhibited by the expression of an artificial micro RNA directed against the *FT *gene (*amiR-FT*) in the phloem companion cells [1,26]. This *amiR-FT *pairs with *FT *transcript in the companion cells of the phloem and stimulates its degradation leading to gene silencing [26]. The *amiR-FT *sequence is complementary to bases 234 to 254 of the *FT *coding sequence (with one mismatch) (Figure 1c).

To overcome the late flowering phenotype of the *amiR-FT *plants, an *FT *orthologue was needed that would not be targeted by the *amiR-FT*, but could function to promote flowering in Arabidopsis. Since, in a related project we are investigating the role of *FT *genes in flowering regulation in the model legume *Medicago truncatula *(Medicago), we investigated whether a Medicago *FT *gene might be a suitable heterologous candidate. There are five *FT *genes in Medicago; the partial genomic DNA sequences of three *FT *genes (*FTa1*, *FTa2 *and *FTc*) were described previously [27], while our database mining revealed full length sequences for these genes and two more, *FTb1 *and *FTb2 *(see also [28,29]). FTa1 is predicted to be the most closely related protein to FT with 71% identity. FTa2, FTb1 and FTb2 share at least 64% identity with FT, while FTc is slightly less similar at 61.7% identity to FT. Two key residues that are important for FT function in Arabidopsis [30,31] are present in four of the predicted Medicago FT proteins (FTa1, FTa2, FTb1 and FTb2) (Figure 1a). The fifth predicted protein, FTc, has the conserved tyrosine residue, but the conserved glutamine in FT is replaced by a histidine (Figure 1a).

Phylogenetic analysis of FT and the related TERMINAL FLOWER1 proteins, that repress flowering, shows that FTa1 and FTa2 form a sister clade to FT/TSF) (Figure 1b). However, although FTa1 shares the highest identity with Arabidopsis FT, all of the FT proteins are as closely related to FT as the known functional FT orthologues, Hd3a and RFT1 from rice [20,21] and SFT from tomato [19].

To test if the Medicago *FTa1 *gene was likely to be targeted by the *amiR-FT*, it was aligned with the *amiR-FT *sequence. No significant similarity was identified using BLAST nucleotide searches. The best alignment that could be made using the MultAlin program is shown in Figure 1c. The number of mismatches in this alignment is 9 over the 21 base sequence, with 5 of these in the 5' region (from bases 2-12), which are important for efficient target-transcript down regulation [26]. This indicated that the *FTa1 *transcript was unlikely to pair with the *amiR-FT *and be degraded.

### Overexpression of *FTa1 *rescues the late flowering phenotype of *amiR-FT *transgenic plants

**Figure 2:**
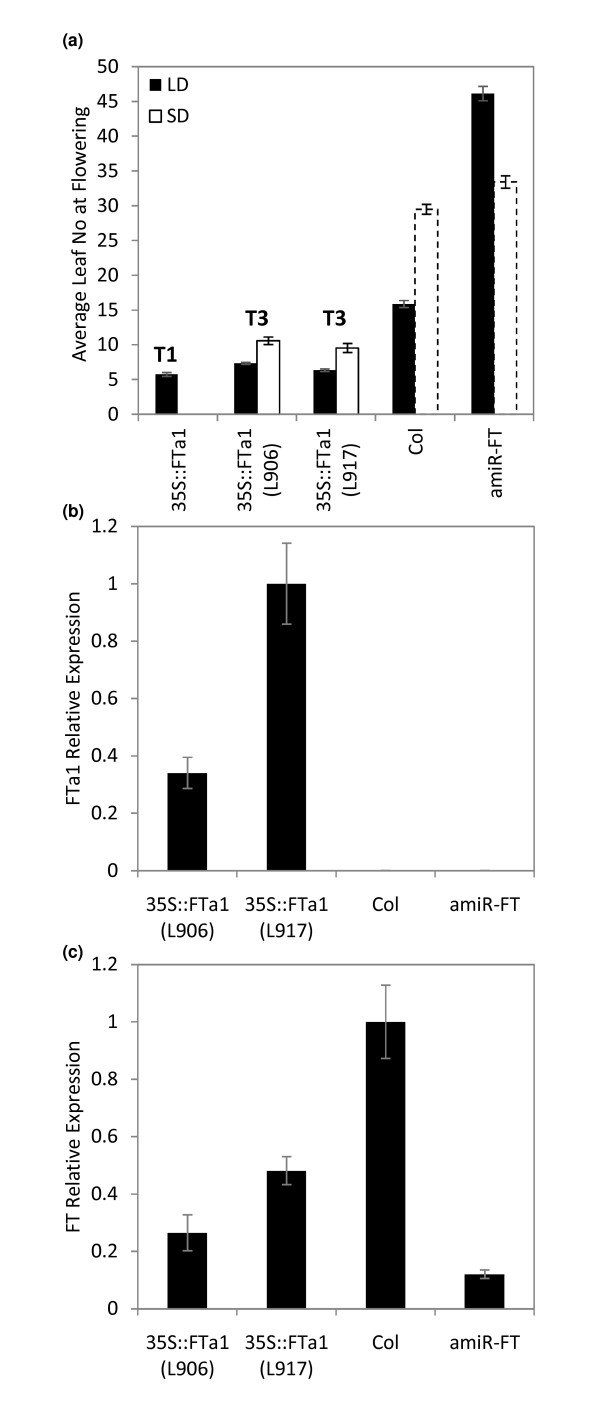
**Overexpression of Medicago *FTa1 *rescues the late flowering phenotype of *amiR-FT *plants**. *AmiR-FT *Arabidopsis transgenic plants were transformed with a *35S::FTa1 *gene expression construct. a) Graph showing average total leaf number at flowering of T1 plants (n = 19), two independent T3 homozygous transgenic lines with the *35S::FTa1 *construct and control plants (n = 11-16). The flowering time of transformant and control Arabidopsis plants in long day (LD) or short day (SD) conditions was measured by scoring the total number of leaves (rosette + cauline) at flowering, unless otherwise specified. The data is presented as mean +/-SE. Nineteen out of 22 of the *35S::FTa1 *T1 transgenic plants flowered earlier than wild-type Columbia (Col) plants; the mean flowering time of these early flowering plants is shown. In the SD experiments, the control Col and *amiR-FT *plants had not yet flowered by 63 days after sowing, at which time the experiment was halted. The leaf number produced by the plants by this time is shown; these bars in the graph are marked with a dashed line b) *FTa1 *transcript accumulation in 10- day-old T3 transgenic lines and control plants in LD was measured using qRT-PCR. Relative transcript abundance of *FTa1 *12 h after lights on in LD conditions is shown with levels normalised to *At2g32170 *(mean +/- SD of 3 PCR replicates is shown). (c) *FT *transcript accumulation in 10- day-old T3 transgenic lines and control plants in LD was measured using qRT-PCR. Relative transcript abundance of *FT *12 h after lights on in LD conditions is shown with levels normalised to *At2g32170 *(mean +/- SD of 3 PCR replicates is shown).

Our first aim was to over express *FTa1 *in the *amiR-FT *plants to test if this would rescue the late flowering phenotype. An expression construct with *FTa1 *fused to the 35S promoter (*35S::FTa1*) was generated. Transformed T1 plants were selected, grown in flowering-inductive long day conditions (LD, 16 h light, 8 h dark) and their flowering time scored (Figure 2a).

Analysis of the leaf number at flowering showed that the majority of the *35S::FTa1 *T1 plants (19/22) flowered considerably earlier than *amiR-FT *plants and wild type control plants, Columbia (Col) (Figure 2a). This result shows that over expression of *FTa1 *promotes flowering in Arabidopsis and fully rescues the late flowering phenotype of *amiR-FT *transgenic plants. Thus, *FTa1 *functions to promote flowering in Arabidopsis, but does not appear to be targeted by the *amiR-FT*.

Before constructing an inducible version of *FTa1*, we confirmed that the early flowering phenotype observed in the T1 generation plants was heritable and expressed in their progeny. Two independent, homozygous T3 transgenic lines with single locus insertions were selected. The T3 transgenic lines were grown in LD or short day (SD, 8 h L/16 h D) conditions. The T3 lines flowered much more rapidly than the control *amiR-FT *plants in both conditions (Figure 2a). These results confirmed that the early flowering trait was heritable. In addition, they showed that *35S::FTa1 *also promoted flowering in non-inductive SD conditions in which flowering of the control Col plants are delayed (Figure 2a).

Expression of the *FTa1 *transgene and endogenous *FT *in the T3 lines and control plants in LD was measured by qRT-PCR (Figure 2b and 2c). Since Arabidopsis *FT *has cyclical diurnal expression, we harvested tissue 12 h after dawn when *FT *levels are rising in wild type plants grown in LD conditions [32]. As expected, in the presence of the *amiR-FT*, *FT *levels were lower in both *FTa1 *transgenic lines and the *amiRNA-FT *line as compared to Col plants (Figure 2c). In contrast, the *FTa1 *transcripts were detected at high levels in the *35S::FTa1 *lines (Figure 2b). This result confirmed that the *FTa1 *transgene was abundantly expressed in the T3 generation plants and this was consistent with their early flowering phenotype.

### Ethanol induces synchronous flowering in *amiR-FT *plants with an *alc::FTa1 *construct

Since expression of *FTa1 *from the 35S promoter rescued the late flowering phenotype of the *amiRNA-FT *lines, we generated an alcohol-inducible version of *FTa1 *(*alc::FTa1*) [33]. *AmiR-FT *Arabidopsis transgenic plants were transformed with the *alc::FTa1 *gene expression construct and 19 T1 transformants were selected. The T2 seed from these *alc::FTa1 *lines were sown out in LD and exposed to ethanol vapour. Eighteen of these T2 families segregated plants that flowered at 20 leaves or less (data not shown) which is much earlier than the *amiRNA-FT *control plants (see Figure 2a). This result suggested that flowering in these lines was inducible by ethanol treatment.

**Figure 3:**
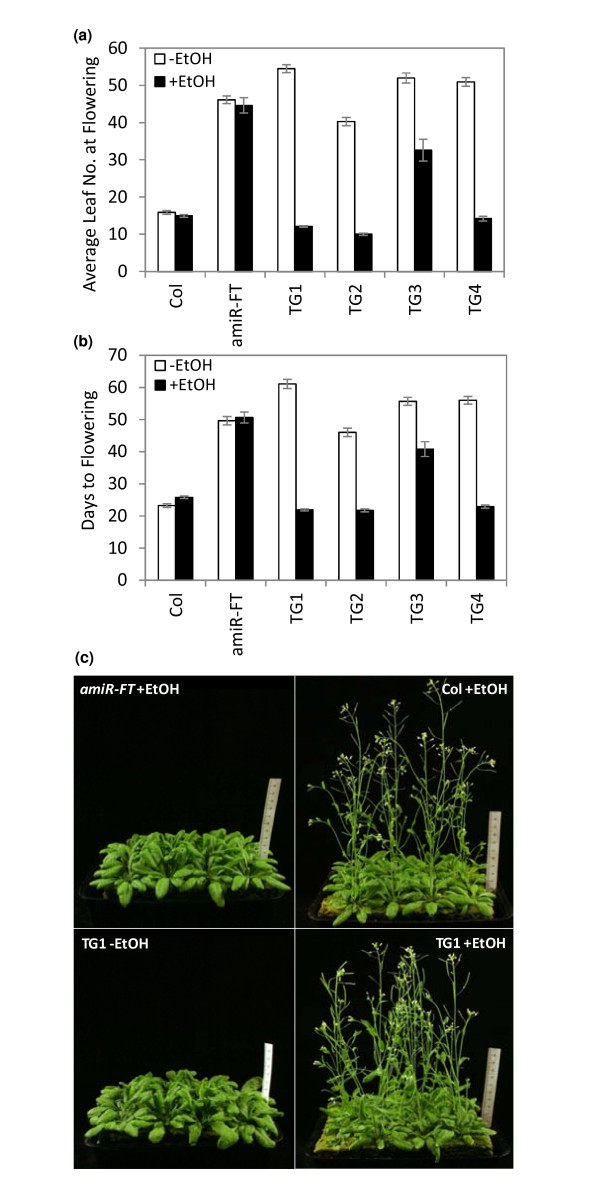
**Ethanol vapour treatments induce flowering in *amiR-FT *plants carrying an alcohol-inducible *FTa1 *gene expression cassette**. *AmiR-FT *Arabidopsis transgenic plants were transformed with an *alc::FTa1 *gene expression construct. Ten day-old *Alc::FTa1 *T3 generation transformants homozygous for single locus insertions (TG1 to TG4) or control plants, in LD conditions, were either exposed (+ETOH), or not (-ETOH), to ethanol vapour for 48 h. Ethanol treatment was repeated 5 days later at day 17, for 24 h. n = 14-16. a) Graph showing average total leaf number at flowering. Flowering time was measured by scoring the total number of leaves (rosette + cauline) at flowering. The data is presented as mean **+/-**SE. b) Graph showing days to flowering. The days to flowering after sowing were scored. The data is presented as mean **+/-**SE. c) Photographs of 39 day-old TG1 transgenic and control plants either exposed (+ETOH), or not (-ETOH), to ethanol vapour.

To confirm this result, four independent homozygous *alc::FTa1 *T3 Lines (TG1 to TG4) with single copy insertions were selected. Plants grown in LD were exposed to dual ethanol vapour treatments in which10 day-old seedlings were treated with ethanol for 48 h and then exposed for a second time at day 17 for 24 h and their flowering time recorded (Figure 3). Three of the TG lines (TG1, 2 and 4) showed strong induction of flowering compared to TG plants grown in the absence of ethanol. Analysis of leaf number at flowering (Figure 3a) shows that the TG plants flowered as early or earlier (TG1, 12.1 +/- SE 0.2; TG2, 10.0 +/- SE 0.3 or TG4, 14.2 +/- SE 0.6 leaves) than wild type Col plants (14.9 +/- SE 0.4 leaves). One other line (TG3) showed a much weaker flowering promotion in response to ethanol (32.6 +/- 2.9 leaves) compared to the -EtOH treatment (52 +/- SE 1.3 leaves). In the absence of ethanol, the TG lines all flowered late (ranging from 40.3 +/- SE 1.1 to 54.5 +/- SE 1.1 leaves) and at a similar time to the *amiR-FT *line (46.1 +/- SE 1.0 leaves). There was no effect of ethanol on flowering of control Col or *amiR-FT *plants.

**Figure 4:**
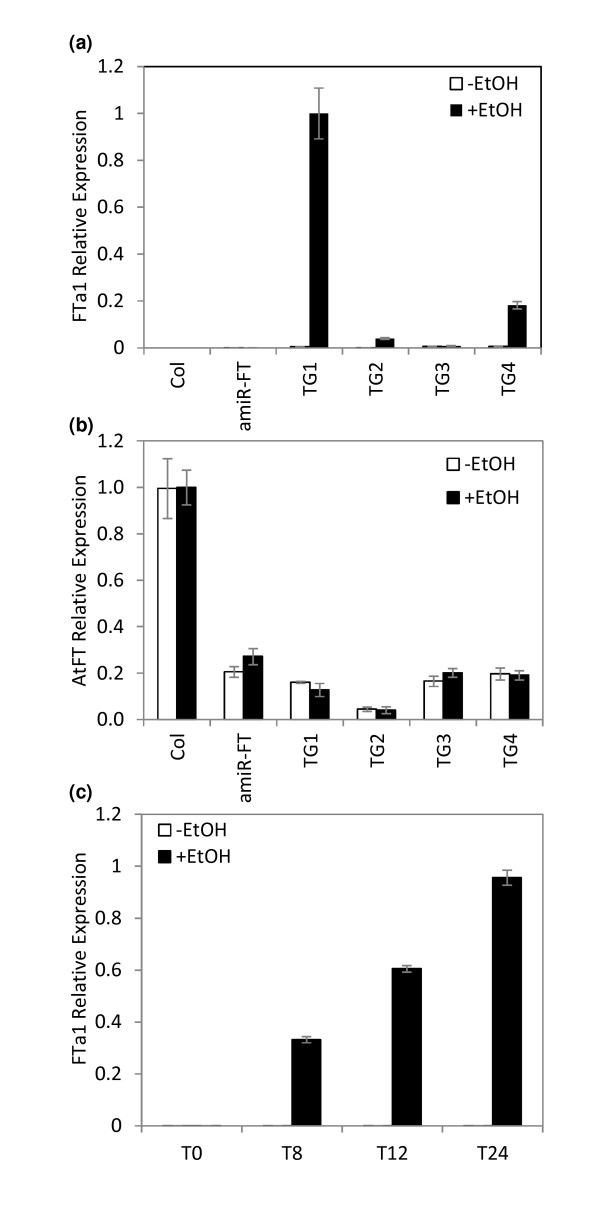
**Induction of *FTa1 *expression by ethanol vapour**. *Alc::FTa1 *T3 transformants or control plants in LD conditions were either exposed (+ETOH), or not (-ETOH), to ethanol vapour. Transcript accumulation in the transgenic lines and control plants was measured using qRT-PCR with levels normalised to *At2g32170*. a) Relative transcript abundance of *FTa1 *after 10-day-old plants (TG1 to TG4 and controls) were exposed to ethanol vapour for 24 h (mean +/- SD of 3 PCR replicates is shown). Induction was started 4 h after dawn and plants were harvested 1 day later at the same time. b) Relative transcript abundance of endogenous Arabidopsis *FT *after ethanol induction as in (a) (mean +/- SD of 3 PCR replicates is shown). c) Time course of induction of *FTa1 *expression by ethanol vapour. Transcript accumulation was measured using qRT-PCR with levels normalised to *At2g32170*. Time course of accumulation of *FTa1 *after 16 day-old TG1 plants were exposed to ethanol vapour for 8, 12 or 24 h (mean +/- SD of qPCR on 2 biological replicates is shown). Induction was started at dawn.

The ability of ethanol to induce *alc::FTa1 *expression was examined using qRT-PCR (Figure 4). Three out of the four TG lines (TG1, 2 and 4) showed good induction of *FTa1 *expression after exposure to 24 h ethanol while one (TG3) showed very little induction (Figure 4a). The very low *FTa1 *induction in TG3 correlated with the delayed flowering in this line (Figure 3a and 3b). However, while TG1, TG2 and TG4 plants flowered at about the same time after induction, the level of *FTa1 *expression was very different. Thus there was an absence of direct correlation between the level of *FTa1 *induction and flowering in the rapid flowering lines. In the untreated TG lines, *FTa1 *was expressed at much lower levels (Figure 4a), but was detectable by qRT-PCR. However, clearly this was not at sufficient levels to overcome the late flowering phenotype conferred by *amiR-FT*. As expected, ethanol treatment did not alter endogenous *FT *accumulation which was expressed at lower levels in the transgenic lines than wild type Col (Figure 4b).

In order to investigate the kinetics of *FTa1 *transcript accumulation, we carried out an induction time course. *FTa1 *expression in TG1 plants in LD in response to ethanol vapour over 24 h was determined (Figure 4c). Levels of *FTa1 *rose strongly within the first 8 h of ethanol exposure and continued to rise over the next 16 h. No change in expression was observed in the untreated TG1 plants over the same time course. This rapid response to ethanol is consistent with previous reports of ethanol induction of *alc*:: reporter gene constructs [33].

These results indicated that ethanol vapour treatments were sufficient to induce *FTa1 *expression and synchronous early flowering and thus rescue the late flowering phenotype of the *amiR-FT *plants carrying the *alc::FTa1 *construct. In addition, the *alc::FTa1 *system gave tight control of the transition to flowering as the transgenic plants flowered late in the absence of ethanol.

### Manipulation of the timing of flowering

**Figure 5:**
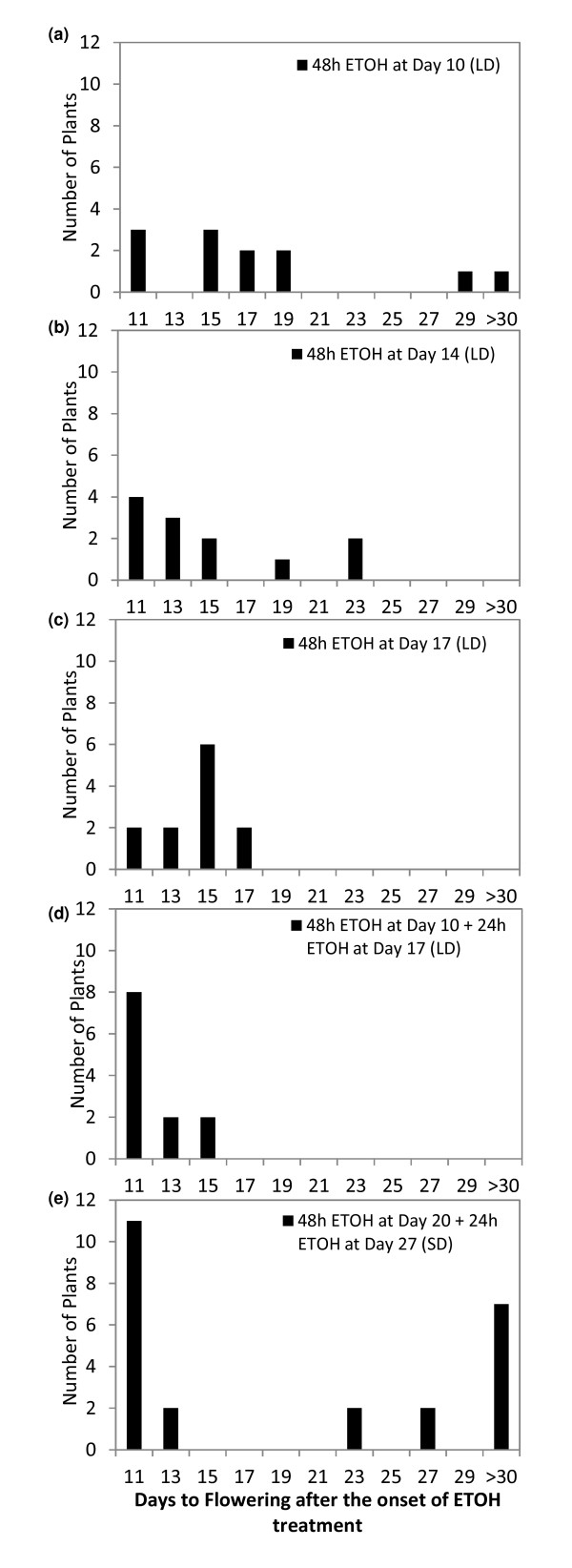
**Manipulation of flowering time**. TG1 *Alc::FTa1 *T3 transformants of different ages growing in LD or SD conditions were exposed to ethanol vapour. The time to flowering measured in days after the start of the ethanol (ETOH) treatment was recorded for each plant. n = 12 for each treatment unless otherwise specified. Plants were grown in LD (a-d) or SD (e). a) Distribution of the time to flowering of 10-day-old plants exposed to ETOH for 48 h. One plant had not yet flowered 30 days after the onset of ETOH treatment. Three out of 12 untreated TG1 plants had flowered by this time (the first visible flower buds were observed 39 days after sowing). b) Distribution of the time to flowering of 14-day-old plants exposed to ETOH for 48 h. c) Distribution of the time to flowering of 17-day-old plants exposed to ETOH for 48 h. d) Distribution of the time to flowering of 10-day-old plants exposed to ETOH for 48 h, followed by a further 24 h treatment when the plants were 17 days old. e) Distribution of the time to flowering of 20-day-old plants in SD conditions exposed to ETOH for 48 h, followed by a further 24 h treatment when the plants were 27 days old. n = 24. Seven plants had not yet flowered 30 days after the onset of ETOH treatment. Floral buds were first observed on 4/24 untreated TG1 plants at this time (50 days after sowing).

Next we tested if the timing of flowering could be manipulated on demand by applying ethanol to plants of different ages. TG1 plants were grown in LD conditions and groups of 10, 14 or 17 day-old plants were exposed to a single 48 h ethanol vapour treatment. We also repeated the dual ethanol treatment of 10-day- old plants as described above. In addition, we tested if flowering could be induced in SD conditions by exposure to ethanol. The number of days to flowering after the onset of each ethanol treatment was recorded for each plant (Figure 5).

In LD conditions, all ethanol treatments resulted in induction of flowering in the majority of plants (Figure 5a-d). Floral buds were first seen on the earliest-flowering plants 11 days after the onset of all the ethanol treatments. Thus these plants flowered 21 days, 25 days or 28 days after sowing. A control group of TG1 plants that were not exposed to ethanol were grown in parallel. The first of these plants (3/12 plants) started to flower 39 days after sowing in LD. Thus the *alc::FTa1 *plants could be induced to flower as early or earlier than wild type Col plants (flowered at ~23 days after sowing, Figure 3b) or later than wild type, by varying the time at which ethanol is applied.

In LD, all of the 14 and 17 day-old-plants were induced to flower more rapidly than the untreated TG1 plants by a single exposure to ethanol (Figure 5b-c). However, the response of the 10-day-old plants to the single ethanol exposure was weaker (Figure 5a). While 10 of these plants were induced to flower by ethanol, the two remaining plants were not. One of these plants flowered 29 days after the start of the treatment (39 days after sowing), while the other still had not flowered by 30 days after the start of treatment (40 days after sowing). This was similar to the untreated TG1 controls.

The most effective of all the treatments in LD was the dual ethanol treatment of the 10-day-old TG1 plants in LD. This resulted in the most synchronous early flowering (Figure 5d). This indicated that there might be the need for more sustained expression of *FTa1*, particularly in younger plants in LD. This may be similar to wild type Arabidopsis plants, where endogenous *FT *is up-regulated by exposing SD-grown plants to a single LD, but three LDs are required for commitment to flowering [34]. In addition, repeated induction of a heat-shock responsive *FT *transgene was most effective at promoting the transition to flowering and ensuing normal flower development in transgenic poplar [24].

Flowering was also able to be induced in the majority of TG1 plants by ethanol in SD conditions (Figure 5e). Twenty day-old SD-grown plants were treated with ethanol for 48 h followed by a 24 h treatment 5 days later. Flower buds were seen 11 days after the onset of ethanol treatment on the earliest flowering plants (31 days after sowing). In LD, the earliest flowering plants also flowered 11 days after the initiation of ethanol treatment (Figure 5a-d). However, not all of the SD plants were induced to flower before the untreated TG1 plants. The latter began to flower from 50 days after sowing (4/24 plants). Seventeen ethanol-treated TG1 plants had flowered before the untreated TG1 controls began to flower, while seven had not (Figure 5e). This indicates that in SD there may be a need for more sustained induction of *FTa1 *or treatment of older plants to obtain floral induction in 100% of plants.

As controls for the induction experiment in SD, we also included ethanol- treated Col and *amiR-FT *plants. None of these plants had flowered by 63 days after sowing, at which time the experiment was halted. In addition, we grew a control alcohol-inducible transgenic line, the *alc::GUS *transgenic line [35]. This gene expression construct allows for ethanol-inducible expression of the *β-GLUCURONIDASE *(*GUS*) reporter gene. None of the *alc::GUS *plants, whether ethanol treated or untreated, had flowered by 63 days after sowing. Thus the early flowering phenotype of the TG1 plants in SD is due to ethanol induction of the *alc::FTa1 *gene-expression cassette.

## Conclusions

Flowering time is a key trait in the breeding of crop and ornamental plants. Our aim was to create a universally-applicable floral induction system that will allow flowering to be induced on demand. Here, we have described such a system and demonstrated its utility in the model plant Arabidopsis.

We showed that the expression of an *FT *orthologue from *Medicago truncatula*, *FTa1*, can overcome the delayed flowering of a transgenic Arabidopsis plants caused by expression of an artificial microRNA to the Arabidopsis *FT *gene, *amiR-FT*. Tight, inducible control of floral induction was engineered successfully using an alcohol-inducible version of the *FTa1 *gene. The timing of flowering could be manipulated by applying ethanol to plants of different ages, to give plants that flowered earlier or later than wild type. Endogenous Arabidopsis *FT *remained low in the transgenic lines, consistent with ongoing silencing by the *amiR-FT*. This indicates that the Medicago *FTa1 *is sufficiently different from Arabidopsis *FT *to escape being targeted by the *amiR-FT*, yet has the ability to strongly promote flowering.

The advantage of the *FT*- based approach tested here in Arabidopsis is that because of the likely universal role of *FT *in triggering of flowering, it should work in other plants. Inducing flowering when desired has many commercial uses [2]. For example, one application of our strategy might be to delay flowering in forage grasses during grazing to give consistent nutritive value and yield, but then later induce synchronous flowering for seed production. Other applications in crops could include inhibiting flowering, followed by inducing flowering to coincide with important market dates, or to avoid peak summer temperatures. The use of the *alc *inducible system should allow the system to be used in a field setting [reviewed by [36]]. Thus, our system has all the attributes required of a biotechnological floral induction system.

Although we have demonstrated the ability to overcome an artificial microRNA that targets a single *FT *gene, in some cultivated species it might be important to down regulate multiple *FT *genes to more effectively delay flowering. For example, Arabidopsis *FT *has the closely-related paralogue *TSF *and double *ft tsf *mutants flower much later than the single mutants [1,11]. In rice, two key *FT *genes have an even stronger impact on flowering as plants with RNAi silencing of both genes had not flowered by 300 days after sowing [21]. Mathieu et al. (2007) demonstrated that it was possible to design an artificial microRNA that could target both the Arabidopsis *FT *and *TSF *mRNA simultaneously. Plants overexpressing this amiR construct phenocopied the late flowering of the *ft tsf *double mutant. Therefore, the use of one or more artifical microRNAs should allow all the *FT *family members involved in the induction of flowering of a particular species to be targeted. Care would then need to be taken to select an *FT *orthologue from another species that would be unaffected by the *amiR*(*s*).

In some cultivated species, natural variants exist that have strongly delayed flowering [2]. In most cases, the genetic reason for the delayed flowering has not been determined. However, it is likely that often this is caused by alterations to pathways upstream of *FT *that prevent or delay the induction of *FT*. Thus, it is an attractive possibility that the late flowering of many natural variants could be utilized in our inducible flowering system so that the targeting of the endogenous *FT *genes using *amiR *would not be necessary and that flowering could be induced simply by using the alcohol-inducible *FT *gene.

In summary, we present proof-of-principle experiments that demonstrate a novel system for inducing flowering on demand, which should provide a biotechnological method for the customization of flowering of commercially important plants.

## Methods

### Database searches, sequence alignments and phylogenetic analysis

*FT *and *TFL1 *genes in *Medicago truncatula *(Medicago) were identified by tBLASTn searches (Basic Local Alignment Search Tool, BLAST) with Arabidopsis FT against Medicago genomic and EST sequences in the National Centre for Biotechnology Information (NCBI) database http://www.ncbi.nlm.nih.gov/blast and the DFCI Medicago Gene Index database http://compbio.dfci.harvard.edu/tgi/cgi-bin/tgi/gimain.pl?gudb=medicago. FT and TFL1 proteins from other species were obtained from GenBank. Protein sequence alignments were performed with ClustalW (^© ^2007 Des Higgins, Julie Thompson, Toby Gibson) and in some cases manually adjusted using the BioEdit Sequence Alignment Editor (^© ^1997-2007 Tom Hall). BioEdit was also used to calculate percentage identity and percentage similarity between a pair of sequences after alignment. After alignment, boot strap analysis with 1000 replications was performed with SEQBOOT. The datasets were then subjected to distance matrix-based phylogenetic analysis using the programs PROTDIST and NEIGHBOR. CONSENSE was used to combine all datasets into one tree based on the majority rule consensus method which only includes groups that are present in more than 50% of the individual trees. All phylogenetic programs were distributed with the Phylogeny Inference Package (PHYLIP) 3.68 (^© ^1980-2008 University of Washington). Phylogenetic trees were displayed with TREEVIEW 1.6.6 (^© ^2000 Roderic D.M. Page).

Gene identifiers or Accession numbers are *AtFT *At1g65480, *AtBFT *At5g62040, *AtTFL1 *At5g03840, *AtTSF *At4g20370, *Gm *TC252514, *HvVRN3 *TC168728, *LeSFT *AY186735, *LeSP *U84140, *LjCEN *AY423715, *MtFTa1 *HQ721813; *MtFTa2 *HQ721814, *MtFTb1 *HQ721815, *MtFTb2 *HQ721816, *MtFTc *HQ721817, *MtTFL1 *TC129531, *OsFDR1 *AF159883, *OsFDR2 *TC304905, *OsHD3a *TC315022, *OsRFT1 *TC315393, *PnFT1 *EU178859, *PnFT2 *EU178860, *PsFT *AY830923, *PsDET *AY340579, *PsLF *AY343326, *PsTFL1b *AY340580, *TaVRN3 *TC322000, *ZmCEN *TC388266.

Alignment of the mRNA sequence of the artificial microRNA to *FT *(*amiR-FT*) with the predicted mRNA of the *FTa1 *gene was done using the MultAlin program [37].

### Plant material, flowering time measurements and ethanol treatments

All plant material used in this work was derived from the *Arabidopsis thaliana *L. Heynh accession Columbia (Col). The *amiR-FT *transgenic line (*SUC2::amiR-FT*; #NW48_1-1) was described previously [1] as was the *alc::GUS *(*AlcAGus*) transgenic line [35]. Flowering time and gene expression analyses were carried out on plants grown under long-day conditions (LD, 16 h light/8 h dark) or short days (SD, 8 h light/16 h dark) in Percival growth cabinets in ~150 μM m^-2 ^s^-1 ^cool white fluorescent light at 22°C in rockwool blocks moistened with hydroponics media [38] [without Na_2_SiO_3_]. Flowering time measurements were carried out by recording the total leaf number at the time of flowering and the days to flowering. Analysis of the flowering time of plants in the presence of ethanol was carried out by exposing plants to ethanol vapour provided by two microfuge tubes each containing 2 ml of 100% ethanol placed at the opposite ends of a rockwool block. The plants and tubes were enclosed using a clear plastic lid (length 38 cm, width 24 cm, height 12 cm) from a Stewart Unheated Propagator which was not airtight. The ethanol regimes used are described in the text. Analysis of gene expression in the presence of ethanol was carried out by exposing plants to ethanol vapour. The regimes used are described in the text.

### Plasmids and plant transformation

A genomic clone with the coding region and introns of the *FTa1 *gene from *Medicago truncatula *were fused to the *CaMV 35S *promoter (*35S::FTa1*) by recombination in the Gateway binary vector PK2GW7 [39], or to the *AlcA *promoter (*alc::FTa1*) in a Gateway compatible *alcR-alcA *binary vector [[33], modified to be Gateway compatible by Lawrence Hobbie and Catherine Perrot-Rechenmann CNRS, Gif sur Yvette, unpublished]. Details of the cloning procedures can be obtained from the authors. The constructs were transformed into *amiR-FT *transgenic plants which were Basta resistant. The kanamycin-resistant T1 transformants were selected in vitro and rescued onto rockwool blocks. Independent homozygous, single copy, T3 lines were bred and used for further work. The presence and identity of the transgenes in the transformed lines were confirmed by PCR and DNA sequencing.

### RNA extraction, cDNA synthesis and qRT-PCR

For gene expression experiments, RNA was extracted from 50 - 100 mg of pooled plant tissue (total aerial parts) using the RNeasy^® ^Plant Mini Kit (Qiagen). A TURBO DNase on-column treatment was carried out after RNA extraction (TURBO DNA-*free*™ Kit, Applied Biosystem). RNA was quantified using a NanoDrop^® ^N-1000 Spectrophotometer (NanoDrop Technologies Inc.). One microgram total RNA was transcribed into cDNA with Superscript III reverse transcriptase (Invitrogen) according to the manufacturer using a (dT)_17 _primer (5'-GACTCGAGTCGACATCGATTTTTTTTTTTTTTTTT-3') [40]. As a control for potential genomic DNA contamination, the same procedure was carried out omitting the reverse transcriptase. To determine relative gene expression levels using quantitative Real Time PCR (qRT-PCR), 2 μl of a 20-fold diluted solution of cDNA was used in a total reaction volume of 10 μl 1× SYBR^® ^Green PCR Master Mix (Applied Biosystems) with final primer concentrations of 0.5 μM. Each cDNA sample was analysed in triplicate PCR reactions, on a 7900 HT Sequence Detection system (Applied Biosystems). Relative gene expression levels were calculated using the 2^-ΔΔCT ^method [41]. The gene expression experiments were repeated on independently grown plants and similar results were obtained. Primers used for quantification of gene expression levels were tested for amplification efficiency prior to use with a dilution series of an arbitrary cDNA sample. The following primer pairs were used for qRT-PCR; *FT*, 5'-CTGGAACAACCTTTGGCA AT-3'and 5'-TACACTGTTTGCCTGCCAAG-3'; *FTa1*, 5' - GTAGCAGTAGGAATCCACTAG C-3' and 5' - ACACTCACTCTCGGTTGATTTCC-3', *At2g32170 *[42], 5'-TGCTTTTTCATCGACACTGC-3' and 5'-CCATATGTGTCCGCAAAATG-3'.

## Authors' contributions

CY carried out flowering time and gene expression experiments and drew the figures, MB carried out database searches, alignments and phylogenetic analysis, drew figures and wrote some of the text, RL and RM provided unpublished materials and results, helped to conceive the study and write the manuscript. JP conceived of the study, supervised the overall project and wrote the manuscript. All authors read and approved the final manuscript.

## References

[B1] Mathieu J Warthmann N Kuttner F Schmid M Export of FT protein from phloem companion cells is sufficient for floral induction in Arabidopsis Current Biology 2007 17 12 1055 1060 10.1016/j.cub.2007.05.009 17540570

[B2] Jung C Muller AE Flowering time control and applications in plant breeding Trends in Plant Science 2009 14 10 563 573 10.1016/j.tplants.2009.07.005 19716745

[B3] Putterill J Laurie R Macknight R It's time to flower: the genetic control of flowering time BioEssays 2004 26 363 373 10.1002/bies.20021 15057934

[B4] Baurle I Dean C The timing of developmental transitions in plants Cell 2006 125 4 655 664 10.1016/j.cell.2006.05.005 16713560

[B5] Mouradov A Cremer F Coupland G Control of flowering time: Interacting pathways as a basis for diversity Plant Cell 2002 14 S111 S130 12045273 10.1105/tpc.001362 PMC151251

[B6] Turck F Fornara F Coupland G Regulation and identity of florigen: FLOWERING LOCUS T moves center stage Annual Review of Plant Biology 2008 59 573 594 10.1146/annurev.arplant.59.032607.092755 18444908

[B7] Kim DH Doyle MR Sung S Amasino RM Vernalization: Winter and the Timing of Flowering in Plants Annual Review of Cell and Developmental Biology 2009 25 277 299 10.1146/annurev.cellbio.042308.113411 19575660

[B8] Kobayashi Y Weigel D Move on up, it's time for change - mobile signals controlling photoperiod-dependent flowering Genes & Development 2007 21 19 2371 2384 10.1101/gad.1589007 17908925

[B9] Kardailsky I Shukla VK Ahn JH Dagenais N Christensen SK Nguyen JT Chory J Harrison MJ Weigel D Activation tagging of the floral inducer *FT* Science 1999 286 1962 1965 10.1126/science.286.5446.1962 10583961

[B10] Kobayashi Y Kaya H Goto K Iwabuchi M Araki T A pair of related genes with antagonistic roles in mediating flowering signals Science 1999 286 1960 1962 10.1126/science.286.5446.1960 10583960

[B11] Yamaguchi A Kobayashi Y Goto K Abe M Araki T *TWIN SISTER OF FT *(*TSF*) acts as a floral pathway integrator redundantly with *FT* Plant and Cell Physiology 2005 46 8 1175 1189 10.1093/pcp/pci151 15951566

[B12] Liu C Thong ZH Yu H Coming into bloom: the specification of floral meristems Development 2009 136 20 3379 3391 10.1242/dev.033076 19783733

[B13] Corbesier L Vincent C Jang SH Fornara F Fan QZ Searle I Giakountis A Farrona S Gissot L Turnbull C FT protein movement contributes to long-distance signaling in floral induction of Arabidopsis Science 2007 316 5827 1030 1033 10.1126/science.1141752 17446353

[B14] Jaeger KE Wigge PA FT protein acts as a long-range signal in Arabidopsis Current Biology 2007 17 12 1050 1054 10.1016/j.cub.2007.05.008 17540569

[B15] Takada S Goto K TERMINAL FLOWER2, a HETEROCHROMATIN PROTEIN1-Like Protein of *Arabidopsis*, counteracts the activation of *FLOWERING LOCUS T *by CONSTANS in the vascular tissues of leaves to regulate flowering time Plant Cell 2003 15 2856 2865 10.1105/tpc.016345 14630968 PMC282816

[B16] Abe M Kobayashi Y Yamamoto S Daimon Y Yamaguchi A Ikeda Y Ichinoki H Notaguchi M Goto K Araki T FD, a bZIP protein mediating signals from the floral pathway integrator FT at the shoot apex Science 2005 309 5737 1052 1056 10.1126/science.1115983 16099979

[B17] Wigge PA Kim MC Jaeger KE Busch W Schmid M Lohmann JU Weigel D Integration of spatial and temporal information during floral induction in Arabidopsis Science 2005 309 5737 1056 1059 10.1126/science.1114358 16099980

[B18] Zeevaart JAD Leaf-produced floral signals Current Opinion in Plant Biology 2008 11 5 541 547 10.1016/j.pbi.2008.06.009 18691931

[B19] Lifschitz E Eviatar T Rozman A Shalit A Goldshmidt A Amsellem Z Alvarez JP Eshed Y The tomato *FT *ortholog triggers systemic signals that regulate growth and flowering and substitute for diverse environmental stimuli Proceedings of the National Academy of Sciences of the United States of America 2006 103 16 6398 6403 10.1073/pnas.0601620103 16606827 PMC1458889

[B20] Kojima S Takahashi Y Kobayashi Y Monna L Sasaki T Araki T Yano M *Hd3a*, a rice ortholog of the *Arabidopsis FT *gene, promotes transition to flowering downstream of *Hd1 *under short-day conditions Plant and Cell Physiology 2002 43 10 1096 1105 10.1093/pcp/pcf156 12407188

[B21] Komiya R Ikegami A Tamaki S Yokoi S Shimamoto K *Hd3a *and *RFT1 *are essential for flowering in rice Development 2008 135 4 767 774 10.1242/dev.008631 18223202

[B22] Tamaki S Matsuo S Wong HL Yokoi S Shimamoto K Hd3a protein is a mobile flowering signal in rice Science 2007 316 5827 1033 1036 10.1126/science.1141753 17446351

[B23] Komiya R Yokoi S Shimamoto K A gene network for long-day flowering activates *RFT1 *encoding a mobile flowering signal in rice Development 2009 136 20 3443 3450 10.1242/dev.040170 19762423

[B24] Zhang HL Harry DE Ma C Yuceer C Hsu CY Vikram V Shevchenko O Etherington E Strauss SH Precocious flowering in trees: the *FLOWERING LOCUS T *gene as a research and breeding tool in Populus Journal of Experimental Botany 2010 61 10 2549 2560 10.1093/jxb/erq092 20406786

[B25] Kotoda N Hayashi H Suzuki M Igarashi M Hatsuyama Y Kidou S Igasaki T Nishiguchi M Yano K Shimizu T Molecular Characterization of *FLOWERING LOCUS T*-*Like *Genes of Apple (*Malus domestica *Borkh) Plant and Cell Physiology 2010 51 4 561 575 10.1093/pcp/pcq021 20189942

[B26] Schwab R Ossowski S Riester M Warthmann N Weigel D Highly specific gene silencing by artificial microRNAs in Arabidopsis Plant Cell 2006 18 5 1121 1133 10.1105/tpc.105.039834 16531494 PMC1456875

[B27] Hecht V Foucher F Ferrandiz C Macknight R Navarro C Morin J Vardy ME Ellis N Beltran JP Rameau C Conservation of Arabidopsis flowering genes in model legumes Plant Physiology 2005 137 4 1420 1434 10.1104/pp.104.057018 15778459 PMC1088331

[B28] Liew LC Hecht V Laurie RE Knowles CL Schoor JKV Macknight RC Weller JL *DIE NEUTRALIS *and *LATE BLOOMER 1 *Contribute to Regulation of the Pea Circadian Clock Plant Cell 2009 21 10 3198 3211 10.1105/tpc.109.067223 19843842 PMC2782296

[B29] Hecht V Laurie RE Vander Schoor JK Ridge S Knowles CL Liew LC Sussmilch FC Murfet IC Macknight RC Weller JL The Pea *GIGAS *Gene Is a *FLOWERING LOCUS T *Homolog Necessary for Graft-Transmissible Specification of Flowering but Not for Responsiveness to Photoperiod Plant Cell 23 1 147 161 10.1105/tpc.110.081042 21282524 PMC3051257

[B30] Ahn JH Miller D Winter VJ Banfield MJ Lee JH Yoo SY Henz SR Brady RL Weigel D A divergent external loop confers antagonistic activity on floral regulators FT and TFL1 Embo Journal 2006 25 3 605 614 10.1038/sj.emboj.7600950 16424903 PMC1383534

[B31] Hanzawa Y Money T Bradley D A single amino acid converts a repressor to an activator of flowering Proceedings of the National Academy of Sciences of the United States of America 2005 102 21 7748 7753 10.1073/pnas.0500932102 15894619 PMC1140427

[B32] Suarez-Lopez P Wheatley K Robson F Onouchi H Valverde F Coupland G *CONSTANS *mediates between the circadian clock and the control of flowering in Arabidopsis Nature 2001 410 6832 1116 1120 10.1038/35074138 11323677

[B33] Roslan HA Salter MG Wood CD White MRH Croft KP Robson F Coupland G Doonan J Laufs P Tomsett AB Characterization of the ethanol-inducible alc gene-expression system in *Arabidopsis thaliana* Plant Journal 2001 28 2 225 235 10.1046/j.1365-313X.2001.01146.x 11722766

[B34] Valverde F Mouradov A Soppe W Ravenscroft D Samach A Coupland G Photoreceptor Regulation of CONSTANS protein in photoperiodic flowering Science 2004 303 1003 1006 10.1126/science.1091761 14963328

[B35] Braun N Wyrzykowska J Muller P David K Couch D Perrot-Rechenmann C Fleming AJ Conditional Repression of AUXIN BINDING PROTEIN1 Reveals That It Coordinates Cell Division and Cell Expansion during Postembryonic Shoot Development in Arabidopsis and Tobacco Plant Cell 2008 20 10 2746 2762 10.1105/tpc.108.059048 18952781 PMC2590743

[B36] Padidam M Chemically regulated gene expression in plants Current Opinion in Plant Biology 2003 6 2 169 177 10.1016/S1369-5266(03)00005-0 12667875

[B37] Corpet F Multiple sequence alignment with hierarchical clustering Nucleic Acids Research 1988 16 10881 10890 10.1093/nar/16.22.10881 2849754 PMC338945

[B38] Gibeaut DM Hulett J Cramer GR Seemann JR Maximal biomass of *Arabidopsis thaliana *using a simple, low-maintenance hydroponic method and favorable environmental conditions Plant Physiology 1997 115 317 319 10.1104/pp.115.2.317 9342857 PMC158488

[B39] Karimi M Inze D Depicker A Gateway vectors for *Agrobacterium*-mediated plant transformation Trends in Plant Science 2002 7 193 195 10.1016/S1360-1385(02)02251-3 11992820

[B40] Frohmann MA Dush MK Martin GR Rapid production of full-length cDNAs from rare transcripts: amplification using a single gene-specific oligonucleotide primer Proceedings of the National Academy of Sciences USA 1988 85 8998 9002 10.1073/pnas.85.23.8998 PMC282649 2461560

[B41] Livak KJ Schmittgen TD Analysis of relative gene expression data using real-time quantitative PCR and the 2-ΔΔCT method Methods 2001 25 402 408 10.1006/meth.2001.1262 11846609

[B42] Czechowski T Stitt M Altmann T Udvardi MK Scheible WR Genome-wide identification and testing of superior reference genes for transcript normalization in Arabidopsis Plant Physiology 2005 139 5 17 10.1104/pp.105.063743 16166256 PMC1203353

